# Supplementary material for: Combining the Benefits of Biotin–Streptavidin Aptamer Immobilization with the Versatility of Ni-NTA Regeneration Strategies for SPR
Source: Sensors (Basel). 2024 Apr 27;24(9):2805. doi: 10.3390/s24092805 (PMC11086168; doi:10.3390/s24092805)
Supplement: Supplementary file 1 [file sensors-24-02805-s001.zip › sensors-2961347-supplementary.pdf]

Table of Contents

Methods – Gel Shift Assay……………………………………………………………………. page 2
Figure S1 – Gel shift assay demonstrating specificity of B-29mer and His-SA binding....... page 3
Figure S2 – Sensorgram demonstrating negligible BSA non-specific binding………..……..page 4
Figure S3 – Full Test Preview of Experiment 3……………………………………………..page 5

Methods - Gel Shift Assay

Aptamers and His-SA from the same sources as Methods section 2.1 were used. Gels were cast in-house from 4% NuSieve GTG Agarose (Lonza, Basel, Switzerland) in 1x Tris-Borate EDTA buffer (VWR, Radnor, PA, USA), and run at 90V for 60 minutes. Aptamers were pre-stained with 1x GelRed nucleic acid stain (Biotium, Fremont, CA, USA). Samples were incubated for 30 minutes at room temperature to allow binding before loading on the gel and running. B-29mer at 1 μM was run alone for reference in lanes 1 and 10. His-SA and B-29mer (both 1 μM) were run in lane 3. B-29mer (1 μM) with 2.4 mg/mL BSA was run in lanes 5 and 6. His-SA (1 μM) and unlabeled 29mer (1 μM) were run in lane 8. Gel was analyzed and photographed on a UV gel imaging box. All aptamers formed a two-band pattern attributable to dimerization. This problem was resolved by heat cycling the aptamer before combining it with protein targets and running the gel shift assay (data not shown). The appearance of a higher weight band in the lane containing B-29mer and His-SA (as well as the lightening of the free aptamer bands) shows the binding between the biotin tagged aptamer and the His-SA. This binding is not seen in lane 8 between the unlabeled aptamer and the His-SA protein. The brightness of the image was reduced to –50 and the contrast was increased to 100 in the Windows Photo app in order to make bands more distinguishable against the background.

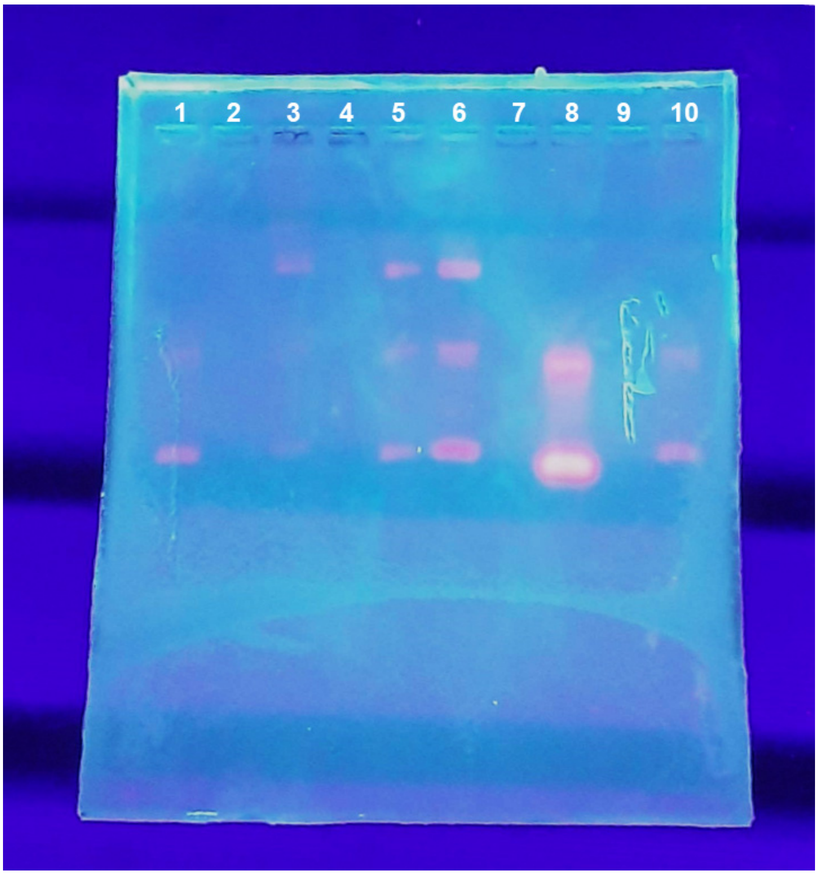

Figure S1: Gel shift assay demonstrating specificity of binding between B-29mer and His-SA protein. B-29mer alone was run in lanes 1 and 10, B-29mer with His-SA in lane 3, B-29mer with BSA in lanes 5 and 6, and unlabeled 29mer with His-SA is shown in lane 8. Photo contrast was adjusted to make bands more distinguishable against the background.

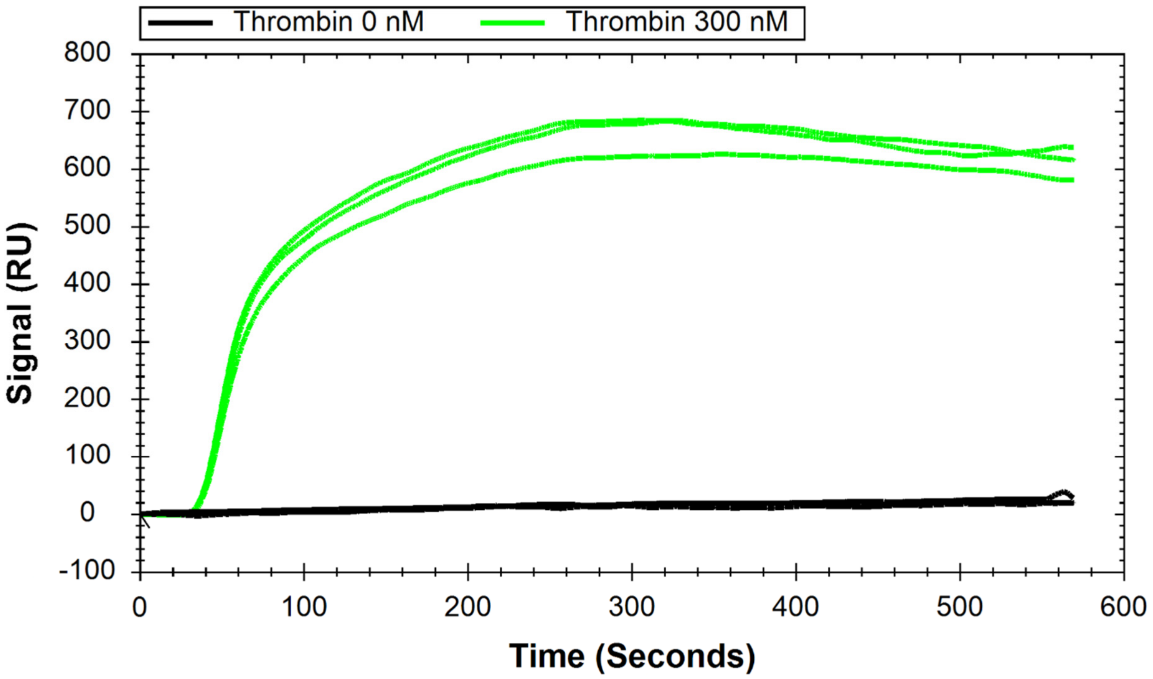

Figure S2: Sensorgram demonstrating low corrected signal for the 0 nM blank sample compared to the highest concentration signal at 300 nM for experiment 3.

The average signals in Ch1 and Ch2 during experiment 3 for the 0 nM sample were –36.02 and –16.33 RU, respectively. The average corrected signal for the 0 nM sample as a percentage of the highest measured binding signal was 3.2% for experiment 3 and 1.3% for experiment 4.

Negligible signal resulted from the binding of BSA protein to immobilized B-29mer and His-SA alone, as seen in the raw Ch1 and Ch2 signals. In addition, the amount of BSA in each sample was held constant.

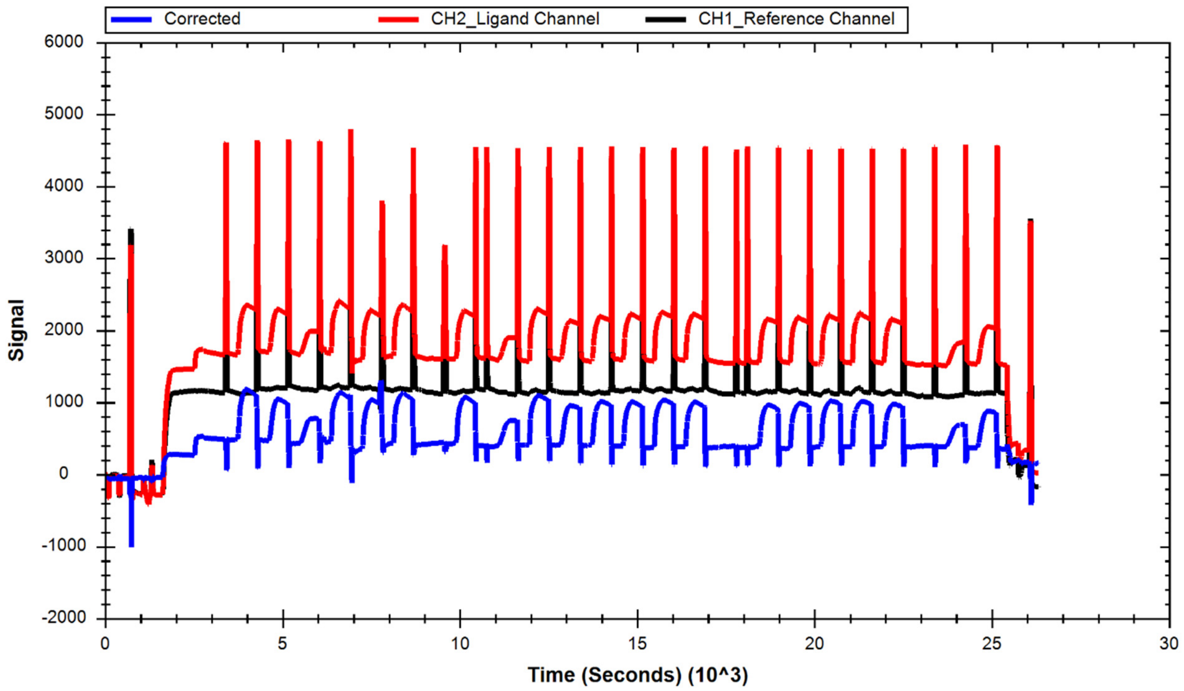

Figure S3: Full Test preview of Experiment 3. A stable baseline can be seen in both channels, indicating successful removal of thrombin protein in between analyte injections.
